# Arthroscopic anterior talofibular ligament repair for lateral instability of the ankle

**DOI:** 10.1007/s00167-015-3638-0

**Published:** 2015-05-16

**Authors:** Masato Takao, Kentaro Matsui, James W. Stone, Mark A. Glazebrook, John G. Kennedy, Stephane Guillo, James D. Calder, Jon Karlsson, Ankle Instability Group

**Affiliations:** Department of Orthopaedic Surgery, Teikyo Institute of Sports Science and Medicine, Teikyo University, 2-11-1 Kaga, Itabashi, Tokyo 173-8605 Japan; Medical College of Wisconsin, Milwaukee, WI USA; Queen Elizabeth II Health Sciences Center Halifax Infirmary (Suite 4867), Dalhousie University, 1796 Summer Street, Halifax, NS B3H 3A7 Canada; Hospital for Special Surgery, East River Professional Building, 523 East 72nd Street, Suite 507, New York, NY 10021 USA; Centre for Orthopaedic Sports Surgery, Bordeaux-Mérignac, France; Chelsea and Westminster Hospital, UK Fortius Clinic, London, UK; Department of Orthopaedics, Sahlgrenska University Hospital, Sahlgrenska Academy, Gothenburg University, 431 80 Mölndal, Sweden

**Keywords:** Lateral instability of the ankle, Arthroscopic repair, Anterior talofibular ligament, Self-cinching stitch, Lasso-loop stitch, Ankle arthroscopy

## Abstract

Although several arthroscopic procedures for lateral ligament instability of the ankle have been reported recently, it is difficult to augment the reconstruction by arthroscopically tightening the inferior extensor retinaculum. There is also concern that when using the inferior extensor retinaculum, this is not strictly an anatomical repair since its calcaneal attachment is different to that of the calcaneofibular ligament. If a ligament repair is completed firmly, it is unnecessary to add argumentation with inferior extensor retinaculum. The authors describe a simplified technique, repair of the lateral ligament alone using a lasso-loop stitch, which avoids additionally tighten the inferior extensor retinaculum. In this paper, it is described an arthroscopic anterior talofibular ligament repair using lasso-loop stitch alone for lateral instability of the ankle that is likely safe for patients and minimal invasive.

*Level of evidence* Therapeutic study, Level V.

## Introduction

Lateral ligament reconstruction of the ankle is indicated when conservative measures have failed to improve functional lateral ankle instability in order to prevent subsequent disorders such as osteochondral lesions of the talar dome and osteoarthritis of the ankle [8–10, 17]. The direct anatomic repair of lateral ligaments of the ankle, originally described by Broström [4], is popular, and the subsequent augmentation of the technique by additionally tightening the inferior extensor retinaculum (IER) has resulted in good outcomes being reported in the literature [11]. However, the open technique requires at least a 4-cm-long incision with significant dissection and soft tissue debridement, and it sometimes causes superficial nerve injury [11].

Recently, several authors have reported good results using an arthroscopy-assisted lateral ligament repair [1, 5–7, 12, 14, 15, 18]. Most of them also attempted to reinforce the construct by using the IER but found that this was both technically difficult and added significant surgery time to the procedure [2]. There is also concern that when using the IER, this is not strictly an anatomical repair since its calcaneal attachment is 10 mm anterior to that of the calcaneofibular ligament (CFL) and this may thus restrict full plantar flexion of the ankle. The need to reinforce lateral ligament repair with the IER is therefore debatable [3].

The lasso-loop stitch is one of the self-cinching stitches [13]. Previous studies have shown that they have superior tissue-holding strength when compared to equivalent non-self-cinching stitches, and it is widely used with good clinical results in shoulder surgery to aid margin convergence in rotator cuff repairs and as traction sutures during stabilization procedure [16]. The lasso-loop stitch is also simple and easy to use in a narrow working space. The authors introduced a lasso-loop stitch to arthroscopic repair of the ATFL alone.

## Technical note

The patient is placed in a supine position on an operating table under spinal or general anaesthesia. It is important keep the ankle elevated above the opposite leg by approximately 20 cm using a leg holder with the foot at the distal edge of the bed in order to allow full dorsiflexion of the ankle. No distraction of the ankle joint was necessary. A 2.7 mm, 30° arthroscope was used with an irrigation pump at 60 mmHg.

**Fig. 1:**
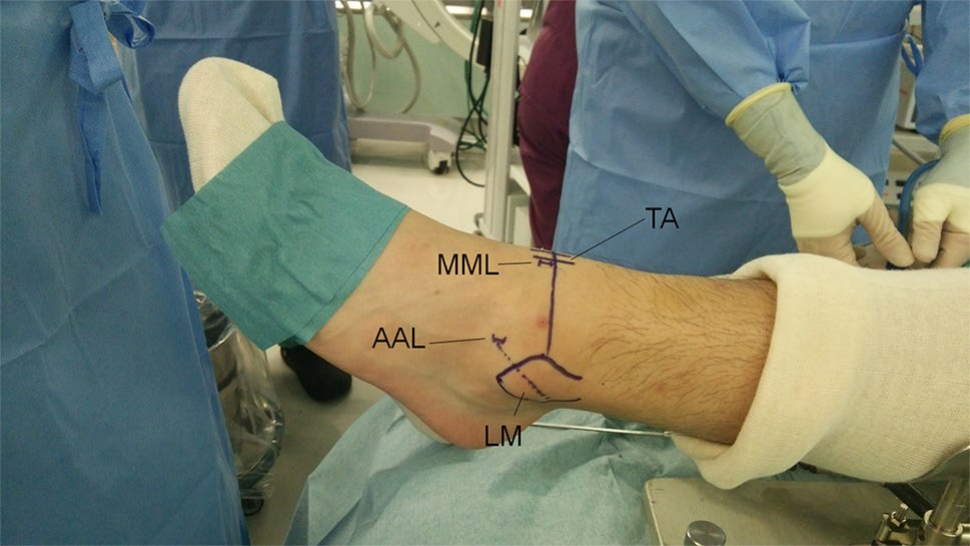
Portals (*MML* medial midline portal, *AAL* accessory anterolateral portal, *TA* tibialis anterior tendon, *LM* lateral malleolus)

Two initial anterior arthroscopy portals were used—the arthroscope was introduced through a medial midline (MML) portal just lateral to the tibialis anterior tendon, and this portal was used to identify the correct position for the accessory anterolateral (AAL) working portal by trans-illumination of the skin (Fig. 1). A needle was used to ensure the correct position of the portal enabling dissection to the fibular ATFL attachment prior to skin incision and blunt dissection with a mosquito clip. Hypertrophic scar tissue was removed where necessary from the lateral gutter using a motorized shaver via the AAL portal, while all other soft tissue including joint capsule and fibula periosteum was left intact.

**Fig. 2:**
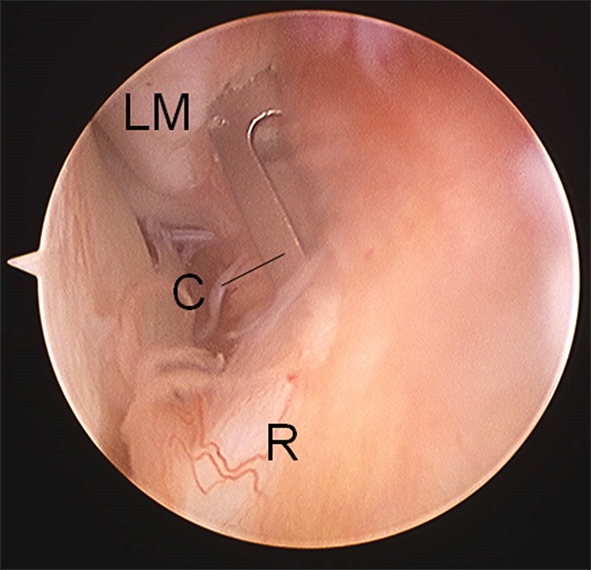
Placing a suture anchor. Arthroscopic view (middle, *LM* lateral malleolus, *C* cannula, *R* remnant of the anterior talofibular ligament)

A suture anchor is placed in the fibula ATFL footprint via the ALL portal. The correct position is ensured by identifying the fibula attachment of the anterior inferior tibiofibular ligament (AITFL) and the drill hole made approximately 7 mm distal to the lower edge of the AITFL. The anchor is then inserted into this hole and leaving the anchor sutures exiting through the ALL portal (Fig. 2).

**Fig. 3:**
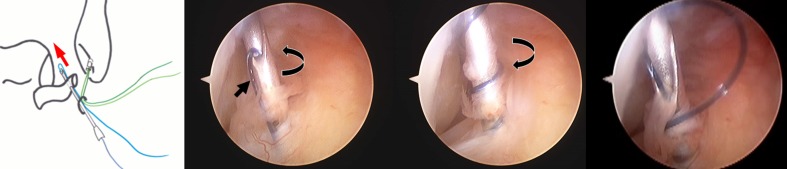
Inserting a needle into distal ATFL remnant. An 18G hollow needle with a 2-0 nylon thread is placed into distal ATFL remnant. The needle is rotated several times one way and then in the opposite direction, enlarging the nylon loop

**Fig. 4:**
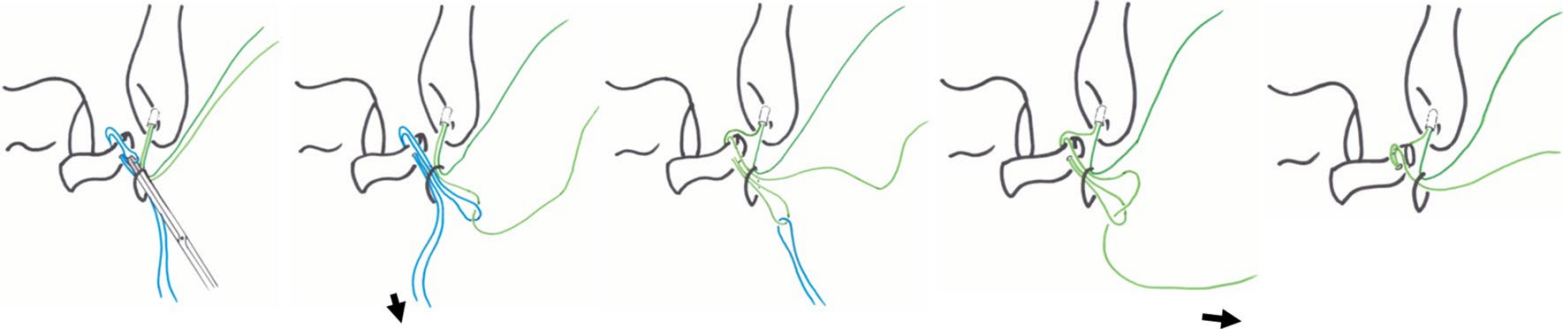
Suture relay technique. The loop of the nylon is retrieved through the ALL portal with a grasping instrument (*left*). One end of the anchor suture is then passed through the loop of nylon (second from the *left*). The nylon loop is then used to pull just the mid-portion of the anchor suture through the ATFL such that a loop of suture is created in the ATFL (*middle*). The free end of the suture is then passed through this loop (second from the *right*) and pulled tight creating a self-clinching stitch (*right*)

**Fig. 5:**
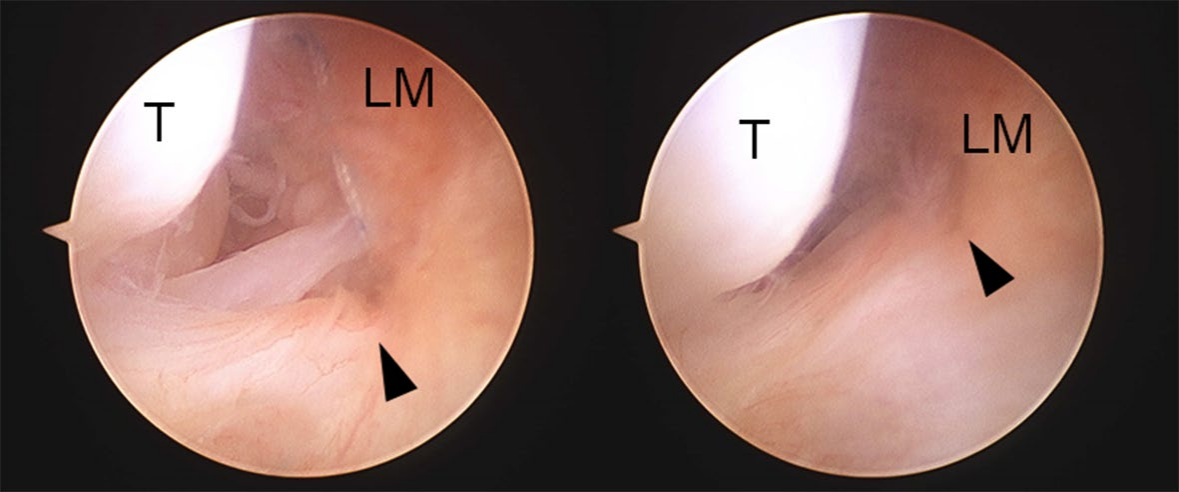
Arthroscopic view. End of a remnant of the ATFL (*left*) is moved to a bone side (*right*) by pulling contralateral thread (arrowhead: End of a remnant of the ATFL, *LM* lateral malleolus)

**Fig. 6:**
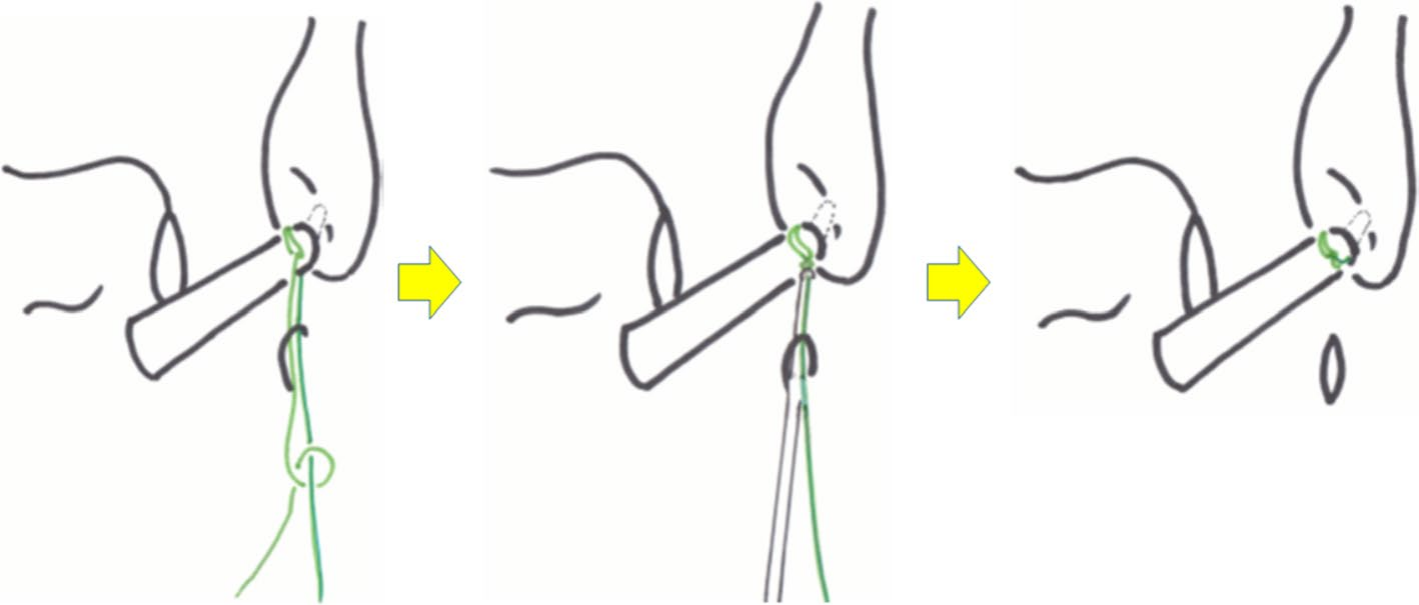
Square knot by turns to an axial thread using a knot pusher

For the lasso-loop stitch, an 18G hollow needle with a 2-0 nylon thread is placed through the ALL portal and into distal ATFL remnant. The needle is rotated several times one way and then in the opposite direction, enlarging the nylon loop (Fig. 3). The loop of the nylon is retrieved through the ALL portal with a grasping instrument, and the needle is then withdrawn. One end of the anchor suture is then passed through the loop of nylon. The nylon loop is then used to pull just the mid-portion of the anchor suture through the ATFL such that a loop of suture is created in the ATFL. The free end of the suture is then passed through this loop and pulled tight creating a self-cinching stitch (Fig. 4). The other end of the anchor suture is then used to draw the self-cinching stitch tightening the ATFL (Fig. 5). Then, square knot and granny knot are done two times using a knot pusher by turns to an axial thread (Fig. 6).

## References

[1] Acevedo JI, Mangone PG (2011). Arthroscopic lateral ankle ligament reconstruction. Tech Foot Ankle Surg.

[2] Aydogan U, Glisson RR, Nunley JA (2006). Extensor retinaculum augmentation reinforces anterior talofibular ligament repair. Clin Orthop Relat Res.

[3] Behrens SB, Drakos M, Lee BJ, Paller D, Hoffman E, Koruprolu S, DiGiovanni CW (2013). Biomechanical analysis of Brostrom versus Brostrom–Gould lateral ankle instability repair. Foot Ankle Int.

[4] Broström L (1966). Sprained ankles. V. Treatment and prognosis in recent ligament ruptures. Acta Chir Scand.

[5] Corte-Real NM, Moreira RM (2009). Arthroscopic repair of chronic lateral ankle instability. Foot Ankle Int.

[6] Cotton JM, Rigby RB (2013). The “all inside” arthroscopic Broström procedure: a prospective study of 40 consecutive patients. J Foot Ankle Surg.

[7] Giza E, Shin EC, Wong SE, Acevedo JI, Mangone PG, Olson K, Anderson MJ (2013). Arthroscopic suture anchor repair of the lateral ligament complex: a cadaver study. Am J Sports Med.

[8] Gross P, Marti B (1999). Risk of degenerative ankle joint disease in volleyball players: study of former elite athletes. Int J Sports Med.

[9] Harrington KD (1979). Degenerative arthritis of the ankle secondary to long-standing lateral ligament instability. J Bone Joint Surg Am.

[10] Hirose K, Murakami G, Minowa T, Kura H, Yamashita T (2004). Lateral ligament injury of the ankle and associated articular cartilage degeneration in the talocrural joint: anatomic study using elderly cadavers. J Orthop Sci.

[11] Karlsson J, Eriksson BI, Bergsten T, Rudholm O, Swärd L (1997). Comparison of two anatomic reconstructions for chronic lateral instability of the ankle joint. Am J Sports Med.

[12] Kim ES, Lee KT, Park JS, Lee YK (2011). Arthroscopic anterior talofibular ligament repair for chronic ankle instability with a suture anchor technique. Orthopedics.

[13] Lafosse L, van Raebroeckx A, Brzoska R (2006). A new technique to improve tissue grip the lasso-loop stitch. Arthroscopy.

[14] Matsui K, Takao M, Miyamoto W, Innami K, Matsushita T (2014). Arthroscopic Broström repair with Gould argumentation via an accessory anterolateral port for lateral instability of the ankle. Arch Orthop Trauma Surg.

[15] Nery C, Raduan F, Buono AD, Asaumi ID, Cohen M, Maffulli N (2011). Arthroscopic-assisted Broström–Gould for chronic ankle instability: a long-term follow-up. Am J Sports Med.

[16] Ponce BA, Hosemann CD, Raghava P, Tate JP, Eberhardt AW, Lafosse L (2011). A biomechanical evaluation of 3 arthroscopic self-cinching stitches for shoulder arthroscopy: the lasso-loop, lasso-mattress, and double-cinch stitches. Am J Sports Med.

[17] Takao M, Ochi M, Uchio Y, Naito K, Kono T, Oae K (2003). Osteochondral lesions of the talar dome associated with trauma. Arthroscopy.

[18] Vega J, Golanó P, Pellegrino A, Rabat E, Peña F (2013). All-inside arthroscopic lateral collateral ligament repair for ankle instability with a knotless suture anchor technique. Foot Ankle Int.

